# Sparse logistic regression with a L_1/2_ penalty for gene selection in cancer classification

**DOI:** 10.1186/1471-2105-14-198

**Published:** 2013-06-19

**Authors:** Yong Liang, Cheng Liu, Xin-Ze Luan, Kwong-Sak Leung, Tak-Ming Chan, Zong-Ben Xu, Hai Zhang

**Affiliations:** 1 Faculty of Information Technology & State Key Laboratory of Quality Research in Chinese Medicines, Macau University of Science and Technology, Macau, China; 2 Department of Computer Science and Engineering, The Chinese University of Hong Kong, Hong Kong, China; 3 Faculty of Science, Xi’an Jiaotong University, Xian, China

**Keywords:** Gene selection, Sparse logistic regression, Cancer classification

## Abstract

**Background:**

Microarray technology is widely used in cancer diagnosis. Successfully identifying gene biomarkers will significantly help to classify different cancer types and improve the prediction accuracy. The regularization approach is one of the effective methods for gene selection in microarray data, which generally contain a large number of genes and have a small number of samples. In recent years, various approaches have been developed for gene selection of microarray data. Generally, they are divided into three categories: filter, wrapper and embedded methods. Regularization methods are an important embedded technique and perform both continuous shrinkage and automatic gene selection simultaneously. Recently, there is growing interest in applying the regularization techniques in gene selection. The popular regularization technique is Lasso (L_1_), and many L_1_ type regularization terms have been proposed in the recent years. Theoretically, the L*q* type regularization with the lower value of *q* would lead to better solutions with more sparsity. Moreover, the L_1/2_ regularization can be taken as a representative of L*q* (0 <*q* < 1) regularizations and has been demonstrated many attractive properties.

**Results:**

In this work, we investigate a sparse logistic regression with the L_1/2_ penalty for gene selection in cancer classification problems, and propose a coordinate descent algorithm with a new univariate half thresholding operator to solve the L_1/2_ penalized logistic regression. Experimental results on artificial and microarray data demonstrate the effectiveness of our proposed approach compared with other regularization methods. Especially, for 4 publicly available gene expression datasets, the L_1/2_ regularization method achieved its success using only about 2 to 14 predictors (genes), compared to about 6 to 38 genes for ordinary L_1_ and elastic net regularization approaches.

**Conclusions:**

From our evaluations, it is clear that the sparse logistic regression with the L_1/2_ penalty achieves higher classification accuracy than those of ordinary L_1_ and elastic net regularization approaches, while fewer but informative genes are selected. This is an important consideration for screening and diagnostic applications, where the goal is often to develop an accurate test using as few features as possible in order to control cost. Therefore, the sparse logistic regression with the L_1/2_ penalty is effective technique for gene selection in real classification problems.

## Background

With the development of DNA microarray technology, the biology researchers can analyze the expression levels of thousands of genes simultaneously. Many studies have demonstrated that microarray data are useful for classification of many cancers. However, from the biological perspective, only a small subset of genes is strongly indicative of a targeted disease, and most genes are irrelevant to cancer classification. The irrelevant genes may introduce noise and decrease classification accuracy. Moreover, from the machine learning perspective, too many genes may lead to overfitting and can negatively influence the classification performance. Due to the significance of these problems, effective gene selection methods are desirable to help to classify different cancer types and improve prediction accuracy.

In recent years, various approaches have been developed for gene selection of microarray data. Generally, they are divided into three categories: filter, wrapper and embedded methods. Filter methods evaluate a gene based on discriminative power without considering its correlations with other genes [1-4]. The drawback of filter methods is that it examines each gene independently, ignoring the possibility that groups of genes may have a combined effect which is not necessarily reflected by the individual performance of genes in the group. This is a common issue with statistical methods such as *T*-test, which examine each gene in isolation.

Wrapper methods utilize a particular learning method as feature evaluation measurement to select the gene subsets in terms of the estimated classification errors and build the final classifier. Wrapper approaches can obtain a small subset of relevant genes and can significantly improve classification accuracy [5,6]. For example, Guyon et al. [7] proposed a gene selection approach utilizing support vector machines (SVM) based on recursive feature elimination. However, the wrapper methods greatly require extensive computational time.

The third group of gene selection procedures is embedded methods, which perform the variable selection as part of the statistical learning procedure. They are much more efficient computationally than wrapper methods with similar performance. Embedded methods have drawn much attention recently in the literature. The embedded methods are less computationally expensive and less prone to over fitting than the wrapper methods [8].

Regularization methods are an important embedded technique and perform both continuous shrinkage and automatic gene selection simultaneously. Recently, there is growing interest in applying the regularization techniques in the logistic regression models. Logistic regression is a powerful discriminative method and has a direct probabilistic interpretation which can obtain probabilities of classification apart from the class label information. In order to extract key features in classification problems, a series of regularized logistic regression methods have been proposed. For example, Shevade and Keerthi [9] proposed the sparse logistic regression based on the Lasso regularization [10] and Gauss-Seidel methods. Glmnet is the general approach for the L_1_ type regularized (including Lasso and elastic net) linear model using a coordinate descent algorithm [11,12]. Similar to sparse logistic regression with the L_1_ regularization method, Gavin C. C. and Nicola L. C. [13] investigated sparse logistic regression with Bayesian regularization. Inspired by the aforementioned methods, we investigate the sparse logistic regression model with a L_1/2_ penalty, in particular for gene selection in cancer classification. The L_1/2_ penalty can be taken as a representative of L*q* (0 <*q* < 1) penalty and has demonstrated many attractive properties, such as unbiasedness, sparsity and oracle properties [14].

In this paper, we develop a coordinate descent algorithm to the L_1/2_ regularization in the sparse logistic regression framework. The approach is applicable to biological data with high dimensions and low sample sizes. Empirical comparisons with sparse logistic regressions with the L_1_ penalty and the elastic net penalty demonstrate the effectiveness of the proposed L_1/2_ penalized logistic regression for gene selection in cancer classification problems.

## Methods

### Sparse logistic regression with the L_1/2_ penalty

In this paper, we focus on a general binary classification problem. Suppose we have *n* samples, *D* = {(*X*_1_, *y*_1_), (*X*_2_, *y*_2_), …, (*X*_*n*_, *y*_*n*_)}, where *X*_*i*_ = (*x*_*i*1_, *x*_*i*2_ , …,  *x*_*ip*_) is *i*^th^ input pattern with dimensionality *p* and *y*_*i*_ is a corresponding variable that takes a value of 0 or 1; *y*_*i*_*=* 0 indicates the *i*^th^ sample in Class 1 and *y*_*i*_*=* 1 indicates the *i*^th^ sample is in Class 2. The vector *X*_*i*_ contains *p* features (for all *p* genes) for the *i*^th^ sample and *x*_*ij*_ denotes the value of gene *j* for the *i*^th^ sample. Define a classifier *f*(*x*) = *e*^*x*^ /(1 + *e*^*x*^) such that for any input *x* with class label *y*, *f*(*x*) predicts *y* correctly. The logistic regression is expressed as:

(1) $$P\left(Y_{i}=1|X_{i}\right)=f\left({X^{\prime }}_{i}\beta \right)=\frac{\text{exp}\left({X^{\prime }}_{i}\beta \right)}{1+\text{exp}\left({X^{\prime }}_{i}\beta \right)}$$

Where *β* = (*β*_*0*_, *β*_*1*_,…, *β*_*p*_) are the coefficients to be estimated, note that *β*_0_ is the intercept. The log-likelihood is:

(2) $$l\left(\beta |D\right)=-\sum_{i=1}^{n}\left\{y_{i}\log\;\left[f\left({X^{\prime }}_{i}\beta \right)\right]+\left(1-y_{i}\right)\log\left[1-f\left({X^{\prime }}_{i}\beta \right)\right]\right\}$$

We can obtain *β* by minimizing the log-likelihood (2). In high dimensional application with *p* >>*n*, directly solving the logistic model (2) is ill-posed and may lead to overfitting. Therefore, the regularization approaches are applied to address the overfitting problem. When adding a regularization term to (2), the sparse logistic regression can be modelled as:

(3) $$\beta =\text{arg}\;\text{min}\left\{l\left(\beta |D\right)+\lambda \sum_{j=1}^{p}P\left(\beta_{j}\right)\right\}$$

Where *λ* > 0 is a tuning parameter and *P*(*B*) is a regularization term. The popular regularization technique is Lasso (L_1_) [10], which has the regularization term *P*(*β*) = ∑ |*β*|. Many L_1_ type regularization terms have been proposed in the recent years, such as SCAD [15], elastic net [16], and MC+ [17].

Theoretically, the L*q* type regularization *P*(*β*) = ∑ |*β*|^*q*^ with the lower value of *q* would lead to better solutions with more sparsity. However when *q* is very close to zero, difficulties with convergence arise. Therefore, Xu et al. [14] further explored the properties of L*q* (0 <*q* <1) regularization and revealed the extreme importance and special role of the L_1/2_ regularization. They proposed that when 1/2<*q* <1, the L_1/2_ regularization can yield most sparse results and its difficulty with convergence is not very high compared with that of the L_1_ regularization, while when 0<*q* <1/2, the performance of L*q* penalties makes no significant difference and solving the L_1/2_ regularization is much simpler than solving the L_0_ regularization. Hence, the L_1/2_ regularization can be taken as a representative of L*q* (0 <*q* < 1) regularizations. In this paper, we apply the L_1/2_ penalty to the logistic regression model. The sparse logistic regression model based on the L_1/2_ penalty has the form:

(4) $$\beta_{1/2}=\text{arg}\;\text{min}\left\{l\left(\beta |D\right)+\lambda \sum_{j=1}^{p}{\left|\beta_{j}\right|}^{1/2}\right\}$$

The L_1/2_ regularization has been demonstrated many attractive properties, such as unbiasedness, sparsity and oracle properties. The theoretical and experimental analyses show that the L_1/2_ regularization is a competitive approach. Our work in this paper also reveals the effectiveness of the L_1/2_ regularization to solve the nonlinear logistic regression problems with a small number of predictive features (genes).

### A coordinate descent algorithm for the L_1/2_ penalized logistic regression

The coordinate descent algorithm [11,12] is a “one-at-a-time” approach, and its basic procedure can be described as follows: for each coefficients, to partially optimize the target function with respect to *β*_*j*_(*j* = 1, 2, …, *p*) with the remaining elements of *β* fixed at their most recently updated values.

Before introducing the coordinate descent algorithm for the nonlinear logistic regularization, we first consider a linear regularization case. Suppose the dataset *D* has *n* samples, *D* = {(*X*_1_, *y*_1_), (*X*_2_, *y*_2_), …, (*Xn*, *y*_*n*_)}, where *X*_*i*_ = (*x*_*i*1_, *x*_*i*2_, …, *x*_*ip*_) is *i*^th^ input variables with dimensionality *p* and *y*_*i*_ is the corresponding response variable. The variables are standardized: $\sum_{i=1}^{n}x_{\mathrm{ij}}^{2}=1$ and $\sum_{i=1}^{n}y_{i}=0.$ Therefore, The linear regression with the regularization term can be expressed as:

(5) $$R\left(\beta \right)=\text{arg}\;\text{min}\left\{\frac{1}{n}\sum_{i=1}^{n}{\left(y_{i}-X^{\prime }\beta \right)}^{2}+\lambda \sum_{j=1}^{p}P\left(\beta_{j}\right)\right\}$$

Where *P*(*B*) is the regularization term. The coordinate descent algorithm solves *β*_*j*_ and other *β*_*k* ≠ *j*_ (*k* ≠ *j* represent the parameters remained after *j*^th^ element is removed) are fixed. The equation (5) can be rewritten as:

(6) $$R\left(\beta \right)=\text{arg}\;\text{min}\left\{\frac{1}{n}{\left(y_{i}-\sum_{k\neq j}x_{\mathrm{ik}}\beta_{k}+x_{\mathrm{ij}}\beta_{j}\right)}^{2}+\lambda \sum_{k\neq j}P\left(\beta_{k}\right)+\mathrm{\lambda P}\left(\beta_{j}\right)\right\}$$

The first order derivative at *β*_*j*_ can be estimated as:

(7) $$\frac{\partial R}{\partial \beta_{j}}=\sum_{i=1}^{n}\left(-x_{\mathrm{ij}}\left(y_{i}-\sum_{k\neq j}x_{\mathrm{ik}}\beta_{k}-x_{\mathrm{ij}}\beta_{j}\right)\right)+\mathrm{\lambda P}\left(\beta_{j}\right)'=0$$

Define ${\tilde{y}}_{i}^{\left(j\right)}=\sum_{k\neq j}x_{\mathrm{ik}}\beta_{k}$ as the partial residual for fitting *β*_*j*_ and $\omega_{j}=\sum_{i=1}^{n}x_{\mathrm{ij}}\left(y_{i}-{\tilde{y}}_{i}^{\left(j\right)}\right)$, the univariate soft thresholding operator of the coordinate descent algorithm [11] for the L_1_ regularization (Lasso) can be defined as:

(8) $$\beta_{j}=S\left(\omega_{j},\lambda \right)=\left\{\begin{array}{cc} \omega_{j}+\lambda & \mathrm{if}\omega_{j}<-\lambda \\ \omega_{j}-\lambda & \mathrm{if}\omega_{j}>\lambda \\ 0 & \mathrm{if}\left|\omega_{j}\right|<\lambda \end{array}\right)$$

Similarly, for the L_0_ regularization, the thresholding operator of the coordinate descent algorithm can be defined as:

(9) $$\beta_{j}=\text{Hard}\left(\omega_{j},\lambda \right)=\mathrm{\omega I}\left(\left|\omega_{j}\right|>\lambda \right)$$

where *I* is the indicator function. This formula is equivalent to the hard thresholding operator [17].

According to equations (8) and (9), we can know that the different penalties are associated with different thresholding operators. Therefore, Xu et al. [18] proposed a half thresholding operator to solve the L_1/2_ regularization for linear regression model. It is an iterative algorithm and can be seen as multivariate half thresholding approach. In this paper, we propose the univariate half thresholding operator of the coordinate descent algorithm for the L_1/2_ regularization. Based on equation (7), the gradient of the L_1/2_ regularization at *β*_*j*_ can be expressed as:

(10) $$\frac{\partial R}{\partial \beta_{j}}=\beta_{j}-\omega_{j}+\lambda \frac{\mathrm{sign}\left(\beta_{j}\right)}{4\sqrt{\left|\beta_{j}\right|}}=0$$

Firstly, we consider the *β*_*j*_ > 0 statement, and let, $\sqrt{\left|\beta_{j}\right|}=\mu$, *β*_*j*_ = *μ*^2^. When *β*_*j*_ > 0, the equation (10) can be redefined as:

(11) $$\mu^{3}-\omega_{j}\mu +\frac{\lambda }{4}=0$$

There are three cases of *ω*_*j*_ < 0, $0<\omega_{j}<\frac{3}{4}\lambda^{\frac{2}{3}}$, and $\omega_{j}>\frac{3}{4}\lambda^{\frac{2}{3}}$ respectively.

(i) If *ω*_*j*_ < 0, the three roots of equation (11) can be expressed as follows:

$$\mu_{1}=-2\;r\text{sin}\;\frac{\varphi }{3},\;\mu_{2}=r\text{sin}\;\frac{\varphi }{3}+i\sqrt{3}\;r\;\text{cos}\;\frac{\varphi }{3}\;\text{and}\;\mu_{3}=r\text{sin}\;\frac{\varphi }{3}-i\;\sqrt{3\;}r\;\text{cos}\;\frac{\varphi }{3},$$

where $r=\sqrt{\frac{\left|\omega_{j}\right|}{3}}$, $\varphi =\text{arccos}\left(\frac{\lambda }{8r^{3}}\right)$. When *r* > 0, none of the roots satisfices *μ*_1_ > 0. Thus, there is no solution to equation (11) when *ω*_*j*_ < 0.

(ii) If $0<\omega_{j}<\frac{3}{4}\lambda^{\frac{2}{3}}$, the three roots of equation (11) are:

$$\mu_{1}=-2\;r\;\text{cos}\;\frac{\varphi }{3},\;\mu_{2}=\;r\;\text{cos}\;\frac{\varphi }{3}+i\;\sqrt{3}r\;\text{sin}\;\frac{\varphi }{3}$$

and $\mu_{3}=r\;\text{cos}\;\frac{\varphi }{3}-i\;\sqrt{3}r\;\text{sin}\;\frac{\varphi }{3}$.

There is still no solution to equation (11) in this case.

(iii) If $\omega_{j}>\frac{3}{4}\lambda^{\frac{2}{3}}$, the three roots of equation (11) are given by:

$$\begin{array}{ll} \cdot \mu_{1} & =-2\;r\;\text{cos}\;\frac{\varphi }{3},\;\cdot \mu_{2}=\;2r\;\text{cos}\;\left(\frac{\pi -\varphi }{3}\right)\;\text{and}\;\mu_{3}\\ & =2r\;\text{cos}\;\left(\frac{\pi +\varphi }{3}\right). \end{array}$$

In this case, the *μ*_2_ is a unique solution of equation (10). Thus, the equation (11) has non-zero roots only when $\omega_{j}>\frac{3}{4}\lambda^{\frac{2}{3}}$. The unique solution of equation (10) is as follow:

$$\beta_{j}={\left(\mu_{2}\right)}^{2}=\frac{2}{3}\left|\omega_{j}\right|\left(1\;+\;\text{cos}\;\left(\frac{2\;\left(\pi \;-\;\varphi \;\left(\omega_{j}\right)\right)}{3}\right)\right)$$

On the other hand, in the *β*_*j*_ < 0 statement, we denoted $\sqrt{\left|\beta_{j}\right|}=\mu$ and *β*_*j*_ = - *μ*^2^. The equation (10) can be transformed into the equation:

(12) $$\mu^{3}-\omega_{j}\mu -\frac{\lambda }{4}=0$$

The equation (12) also has a unique solution when $\omega_{j}<-\frac{3}{4}\lambda^{\frac{2}{3}}$:

$$\mu_{2}=2r\text{cos}\left(\frac{\pi -\varphi }{3}\right)$$

and $\beta_{j}=\;-{\left(\mu_{2}\right)}^{2}=\;-\frac{2}{3}\left|\omega_{j}\right|\left(1\;+\;\text{cos}\;\left(\frac{2\left(\pi -\varphi \left(\omega_{j}\right)\right)}{3}\right)\right)$.

In conclusion, the univariate half thresholding operator can be expressed as:

(13) $$\begin{array}{ll} \beta_{j} & =\text{Half}\left(\omega_{j},\lambda \right)\\ & =\left\{\begin{array}{cc} \frac{2}{3}\omega_{j}\left(1+\text{cos}\left(\frac{2\left(\pi -\varphi_{\lambda }\left(\omega_{j}\right)\right)}{3}\right)\right) & \mathrm{if}\left|\omega_{j}\right|>\frac{3}{4}{\left(\lambda \right)}^{\frac{2}{3}}\\ 0 & \text{otherwise} \end{array}\right) \end{array}$$

where *φ*_*λ*_(*ω*) satisfies:

$$\text{cos}\left(\varphi_{\lambda }\left(\omega \right)\right)=\frac{\lambda }{8}{\left(\frac{\left|\omega \right|}{3}\right)}^{-\frac{3}{2}}$$

The coordinate descent algorithm for the L_1/2_ regularization makes repeated use of the univariate half thresholding operator. The details of the algorithm will be described later. This coordinate descent algorithm for the regularization can be extended to the sparse logistic regression model. Based on the objective function (3) of the sparse logistic regression, one-term Taylor series expansion for *l*(*B*) has the form of

(14) $$L\left(\beta ,\lambda \right)\approx \frac{1}{2n}\sum_{i=1}^{n}\left(Z_{i}-X_{i}\beta \right)'W_{i}\left(Z_{i}-X_{i}\beta \right)+\sum_{j=1}^{p}P\left(\beta_{j}\right)$$

Where $Z_{i}=X_{i}\tilde{\beta }+\frac{Y_{i}-f\left(X_{i}\tilde{\beta }\right)}{f\left(X_{i}\tilde{\beta }\right)\left(1-f\left(X_{i}\tilde{\beta }\right)\right)}$ is an estimated response, $W_{i}=f\left(X_{i}\tilde{\beta }\right)\left(1-f\left(X_{i}\tilde{\beta }\right)\right)$ is a weight and $f\left(X_{i}\tilde{\beta }\right)=\text{exp}\left(X_{i}\tilde{\beta }\right)/\left(1+\text{exp}\left(X_{i}\tilde{\beta }\right)\right)$ is a evaluated value at current parameters. Redefine the partial residual for fitting current ${\tilde{\beta }}_{j}$ as ${\tilde{Z}}_{i}^{\left(j\right)}=\sum_{i=1}^{n}W_{i}\left({\tilde{Z}}_{i}-\sum_{k\neq j}x_{\mathrm{ik}}{\tilde{\beta }}_{k}\right)$ and $\sum_{i=1}^{n}x_{\mathrm{ij}}\left(Z_{i}-{\tilde{Z}}_{i}^{\left(j\right)}\right)$, we can directly apply the coordinate descent algorithm with the L_1/2_ penalty for sparse logistic regression and the details are given follows:

**Algorithm**: The coordinate descent algorithm for sparse logistic with the L_1/2_ penalty

The coordinate descent algorithm for the L_1/2_ penalized logistic regression works well in the sparsity problems, because the procedure does not need to change many irrelevant parameters and recalculate partial residuals for each update step.

## Results

### Analyses of simulated data

In this section, we evaluate the performance of the sparse logistic regression with the L_1/2_ penalty in simulation study. We generate high-dimensional and low sample size data which contain many irrelevant features. Two methods are compared with our proposed approach: Sparse logistic regression with the Elastic Net penalty (L_EN_) and Sparse logistic regression with the Lasso penalty (L_1_).

We generated the vectors *γ*_*i*0_,*γ*_*i*1_,…,*γ*_*ip*_ (*i* = 1,…,*n*) independently from the standard normal distribution and the predictor vector(*i*=1,…,*n*) is generated by $x_{\mathrm{ij}}=\gamma_{\mathrm{ij}}\sqrt{1-\rho }+\gamma_{i0}\sqrt{\rho }$ (*j*=1,…, *p*), where *ρ* is the correlation coefficient of the predictor vectors [19]. The simulated data set generated from the logistic model:

(15) $$\log\left(\frac{Y_{i}}{1-Y_{i}}\right)=\beta_{0}+\sum_{j=1}^{p}x_{\mathrm{ij}}\beta_{j}+\sigma \cdot \epsilon$$

Where *ϵ* is the independent random error generated from *N*(0,1) and *σ* is the parameter which controls the signal to noise. In every simulation, the dimension *p* of the predictor vector is 1000, and the first five true coefficients are nonzero: *β*_1_ = 1, *β*_2_ = 1, *β*_3_ = -1, *β*_4_ = -1, *β*_5_ = 1, and *β*_*j*_ = 0(6 ≤ *j* ≤ 1000).

The estimation of the optimal tuning parameter *λ* in the sparse logistic regression models can be done in many ways and is often done by *k*-fold cross-validation (CV). Note that the choice of *k* will depend on the size of the training set. In our experiments, we use 10-fold cross-validation (*k*=10). The elastic net method has two tuning parameters, we need to cross-validate on a two-dimensional surface [16].

We consider the cases with the training sample size *n* = 50, 80, 100, the correlation coefficient *ρ* =0.1, 0.4 and the noise control parameter *σ* =0.2, 0.6 respectively. Each classifier was evaluated on a test data set including 100 samples. The experiments were repeated 30 times and we report the average test errors in Table 1. As shown in Table 1, when the sample size *n* increases, the prediction performances of all the three methods are improved. For example when *ρ* =0.1, and *σ* =0.2, the average test errors of the L_1/2_ method are 28.2%, 10.7% and 8.1% with the sample sizes *n*=50, 80, and 100 respectively. When the correlation parameter *ρ* and the noise parameter *σ* increase, the prediction performances of all the three methods are decreased. For example, when *ρ* =0.4 and *n* =100, the average test errors from the L_1/2_ method increased from 9.1% to 15.1%, in which *σ* increased from 0.2 to 0.6. When *σ* =0.6 and *n* =80, the average test error from the L_1/2_ method increase from 18.4% to 20.5%, in which *ρ* increased from 0.1 to 0.4. Moreover, in our simulation, the influence of the noise may be larger than that of the variable correlation for the prediction performance of all the three methods. On the other hand, at the same parameter setting case, the prediction performance of the L_1/2_ method is consistent and better than the results of the L_EN_ and L_1_ methods. For example, when*ρ* =0.1, *σ* =0.2 and *n*=100, the predictive error of the L_1/2_ method is 8.1% much better than 16.9% and 15.7% got by the L_EN_ and L_1_ methods respectively.

**Table 1:** **The average errors (%) for the test data sets obtained by the sparse logistic regressions with the L**_**1/2**_**, L**_**EN **_**and L**_**1 **_**penalties in 30 runs**

	**Sample size**	**L**_**1/2**_	**L**_**EN**_	**L**_**1**_
$$\begin{array}{c} \rho =0.1,\\ \sigma =0.2 \end{array}$$	*n*=50	28.2	31.8	31.2
	*n=*80	10.7	23.1	22.2
	*n=*100	8.1	16.9	15.7
$$\begin{array}{c} \rho =0.1,\\ \sigma =0.6 \end{array}$$	*n=*50	31.4	33.1	33.3
	*n=*80	18.4	27.1	26.6
	*n=*100	14.2	22.4	21.3
$$\begin{array}{c} \rho =0.4,\\ \sigma =0.2 \end{array}$$	*n=*50	30.1	32.6	33.0
	*n=*80	11.1	23.3	22.9
	*n=*100	9.1	19.0	16.4
$$\begin{array}{c} \rho =0.4,\\ \sigma =0.6 \end{array}$$	*n=*50	35.1	35.5	36.3
	*n=*80	20.5	27.2	26.9
	*n=*100	15.1	22.7	22.9

Table 2 shows the average number of the variables selected in 30 runs for each method. Since the simulation datasets have *x*_1_-*x*_5_ relevant features, the idealized average number of variables selected by each method is 5. In Table 2, the results obtained by the L_1/2_ penalized method are obviously closed to 5 and 3–10 times smaller than those of the L_EN_ and L_1_ penalties at the same parameter setting. For example, when *ρ* =0.1, *σ =*0.2 and *n*=100, the average numbers from the L_EN_ and L_1_ methods are 49.7 and 45.7 respectively, and the result of L_1/2_ method is 8.9. Moreover, when the sample size *n*, the correlation parameter *ρ*, and the noise parameter *σ* increase, the average numbers from all the three methods increase, but the values of the L_EN_ and L_1_ methods increase faster than those of the L_1/2_ method. This means that the L_1/2_ penalized method consistently outperforms than other two methods in term of variable selection.

**Table 2:** **The average number of variables selected by the sparse logistic regressions with the L**_**1/2**_**, L**_**EN **_**and L**_**1 **_**penalties in 30 runs**

	**Sample size**	**L**_**1/2**_	**L**_**EN**_	**L**_**1**_
$$\begin{array}{c} \rho =0.1,\\ \sigma =0.2 \end{array}$$	*n*=50	7.5	31.6	27.1
	*n=*80	8.8	43.1	40.3
	*n=*100	8.9	49.7	45.7
$$\begin{array}{c} \rho =0.1,\\ \sigma =0.6 \end{array}$$	*n=*50	8.3	33.6	29.2
	*n=*80	10.6	45.7	41.9
	*n=*100	10.8	54.4	50.1
$$\begin{array}{c} \rho =0.4,\\ \sigma =0.2 \end{array}$$	*n=*50	7.8	33.5	28.3
	*n=*80	8.9	44.5	41.8
	*n=*100	9.0	51.2	46.6
$$\begin{array}{c} \rho =0.4,\\ \sigma =0.6 \end{array}$$	*n=*50	8.6	41.3	29.9
	*n=*80	10.7	45.9	44.1
	*n=*100	11.2	56.4	53.4

To further evaluate the performance of the L_1/2_ penalized method, we report the frequency with which each relevant variable was selected among 30 runs for each method in Table 3. When the sample size is small (*n*=50), the L_1/2_ penalty selects the relevant variables slightly less frequently than the other two methods and all the three methods select true nonzero coefficients with difficulties, especially when *ρ* and *σ* are relatively large. For example, when *ρ* =0.4, *σ =*0.6, *n*=50, and for *β*_5_, the selected frequencies of the L_1/2_, L_EN_ and L_1_ methods are 12, 14 and 13 respectively in 30 runs. As *n* increases, all the three methods tend to select the true nonzero coefficients more accurately and the L_1/2_ penalty method performs slightly better, in terms of variable frequencies, than the other two methods under the different parameter settings of *ρ* and *σ.* To sum up, Tables 1, 2 and 3 clearly show that the L_1/2_ method is winner among the competitors in terms of both prediction accuracy and variable selection in the different variable correlation and noise situations.

**Table 3:** **The frequencies of the relevant variables obtained by the sparse logistic regressions with the L**_**1/2**_**, L**_**EN **_**and L**_**1 **_**penalties in 30 runs**

	**Sample size**	**Method**					
$$\begin{array}{c} \rho =0.1,\\ \sigma =0.2 \end{array}$$	*n*=50	L_1/2_	21	22	19	15	15
		L_EN_	24	25	21	17	17
		L_1_	22	24	20	15	17
	*n*=80	L_1/2_	30	30	30	30	30
		L_EN_	30	29	30	30	30
		L_1_	30	29	30	30	30
	*n*=100	L_1/2_	30	30	30	30	30
		L_EN_	30	30	30	30	30
		L_1_	30	30	30	30	30
$$\begin{array}{c} \rho =0.1,\\ \sigma =0.6 \end{array}$$	*n*=50	L_1/2_	17	17	17	14	14
		L_EN_	18	19	17	16	14
		L_1_	18	18	18	16	15
	*n*=80	L_1/2_	30	29	30	28	28
		L_EN_	30	28	30	28	27
		L_1_	30	28	30	27	26
	*n*=100	L_1/2_	30	30	30	30	30
		L_EN_	30	30	30	30	30
		L_1_	30	30	30	28	30
$$\begin{array}{c} \rho =0.4,\\ \sigma =0.2 \end{array}$$	*n*=50	L_1/2_	19	18	18	16	15
		L_EN_	21	22	21	17	17
		L_1_	18	21	19	16	17
	*n*=80	L_1/2_	30	30	30	30	30
		L_EN_	30	28	30	29	29
		L_1_	30	27	30	29	29
	*n*=100	L_1/2_	30	30	30	30	30
		L_EN_	30	30	30	30	30
		L_1_	30	30	30	29	29
$$\begin{array}{c} \rho =0.4,\\ \sigma =0.6 \end{array}$$	*n*=50	L_1/2_	14	16	15	12	12
		L_EN_	17	17	17	12	14
		L_1_	17	15	14	9	13
	*n*=80	L_1/2_	29	25	26	28	29
		L_EN_	28	24	24	27	24
		L_1_	27	24	24	23	23
	*n*=100	L_1/2_	30	29	30	30	30
		L_EN_	30	27	28	28	30
		L_1_	29	27	27	28	30

### Analyses on microarray data

In this section, we compare our proposed L_1/2_ penalized method with the L_EN_ and L_1_ methods on 4 publicly available gene expression datasets: Leukaemia, Prostate, Colon and DLBCL. A brief description of these datasets is given below and summarized in Table 4.

**Table 4:** Four publicly available gene expression datasets used in the experiments

**Dataset**	**No. of genes**	**No. of samples**	**classes**
Leukaemia	3571	72	ALL/AML
Prostate	5966	102	Normal/Tumor
Colon	2000	62	Normal/Tumor
DLBCL	6285	77	DLBCL/FL

### Leukaemia dataset

The original dataset was provided by Golub et al. [7], and contains the expression profiles of 7,129 genes for 47 patients of acute lymphoblastic leukaemia (ALL) and 25 patients of acute myeloid leukaemia (AML). For data preprocessing, we followed the protocol detailed in the supplementary information to Dudoit et al. [1]. After thresholding, filtering, applying a logarithmic transformation and standardizing each expression profile to zero mean and unit variance, a dataset comprising 3,571 genes remained.

### Prostate dataset

This original dataset contains the expression profiles of 12,600 genes for 50 normal tissues and 52 prostate tumor tissues. For data preprocessing, we adopt the pretreatment method [20] to obtain a dataset with 102 samples. And each sample contains 5966 genes.

### Colon dataset

The colon microarray data set in Alon et al. [21] has 2000 genes per sample and 62 samples which consist of 22 normal tissues and 40 cancer tissues. The Colon dataset are available at http://microarray.princeton.edu/oncology.

### DLBCL dataset

This dataset contains 77 microarray gene expression profiles of the 2 most prevalent adult lymphoid malignancies: 58 samples of diffuse large B-cell lymphomas (DLBCL) and 19 observations of follicular lymphoma (FL). Each sample contains 7,129 gene expression values. More information on these data can be found in Shipp MA et al. [22]. For data preprocessing, we followed the protocol detailed in the supplementary information to Dudoit et al. [1], and a dataset comprising 6,285 genes remained.

We evaluate the prediction accuracy of the three penalized logistic regression models using random partition. This means that we divide the datasets at random such that approximate 70-80% of the datasets becomes training samples and the other 20-30% test samples. More information on these data is given in Table 5. For selecting the tuning parameter *λ*, we employ the ten-fold cross validation scheme using the training set. We repeat this procedure 30 times and the averaged misclassification errors were reported in Table 6. Here the denominators of the ten-fold cross validation errors and the test errors describe the sample size of training and test datasets respectively. The fractions of the ten-fold cross validation errors and the test errors and the number of gene selected are the approximated integers of the corresponding average number at 30 runs. As shown in Table 6, for Leukaemia dataset, the classifier with the L_1/2_ penalty gives the average ten-fold cross validation error of 2/50 and the average test error of 1/22 with about 2 genes selected. The classifiers with L_EN_ and L_1_ methods give the average ten-fold cross validation errors of 1/50 and the average test errors of 1/22 with about 9 and 6 genes selected respectively. This means that all three methods can be successfully applied to high-dimensional classification problems and classify the Leukaemia dataset with same accuracies. Note that, the L_1/2_ method achieved its success using only about 2 predictors (genes), compared to about 9 and 6 for the L_EN_ and L_1_ methods. For Prostate and Colon datasets, it can be seen the L_1/2_ method achieves the best classification performances with the highest accuracy rates using much fewer genes compared with those of the L_EN_ and L_1_ methods. For DLBCL dataset, the L_1/2_ logistic regression achieves better classification performance than that of the L_1_ method and worse than that of the L_EN_ method. However, as well as other three datasets, the L_1/2_ method achieved its success using much less predictors (about 14 genes), compared to about 38 and 23 for the L_EN_ and L_1_ methods. This is an important consideration for screening and diagnostic applications, where the goal is often to develop an accurate test using as few features as possible in order to control cost.

**Table 5:** The detail information of 4 microarray datasets used in the experiments

**Dataset**	**No.of Training(class1/class2)**	**No.of Testing(class1/class2)**
Leukaemia	50(32 ALL/18 AML)	22 (15 ALL/7 AML)
Prostate	71(35 ALL/36 AML)	31(15 ALL/16 AML)
Colon	42(14 Normal/28 Tumor)	20(8 Normal/12 Tumor)
DLBCL	60(45 DLBCL/15FL)	17(13 DLBCL/4 FL)

**Table 6:** The classification performances of different methods for 4 gene expression datasets

**Dataset**	**Method**	**Cross-validation error**	**Test error**	**No. of selected genes**
Leukaemia	L_1/2_	2/50	1/22	2
	L_EN_	1/50	1/22	9
	L_1_	1/50	1/22	6
Prostate	L_1/2_	5/71	3/31	5
	L_EN_	5/71	4/31	34
	L_1_	5/71	3/31	25
Colon	L_1/2_	4/42	3/20	5
	L_EN_	5/42	4/20	13
	L_1_	5/42	4/20	7
DLBCL	L_1/2_	3/60	2/17	14
	L_EN_	2/60	1/17	38
	L_1_	3/60	3/17	23

**Figure 1:**
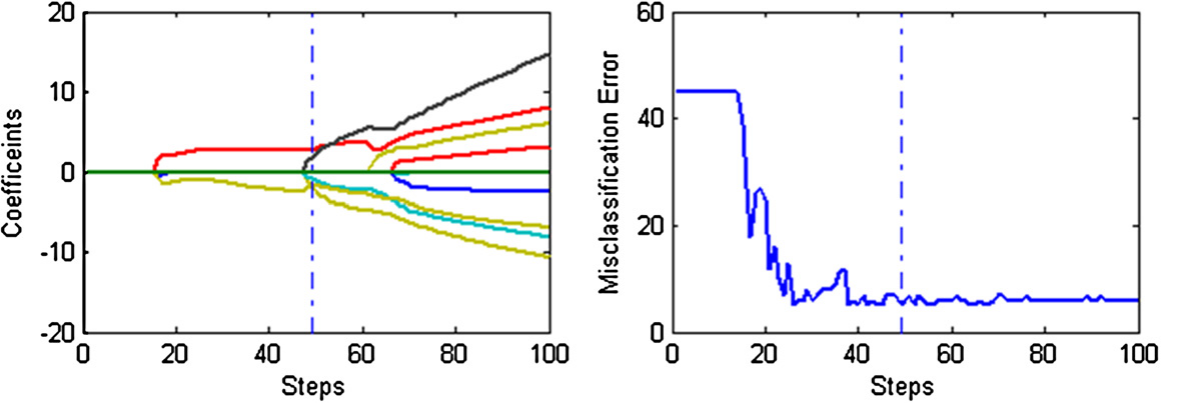
**The results of the sparse logistic regression with the L**_**1/2 **_**penalty on Prostate dataset.** The solution paths and the gene selection results of the sparse logistic L_1/2_ penalty methods for the Prostate dataset in one sample run.

**Figure 2:**
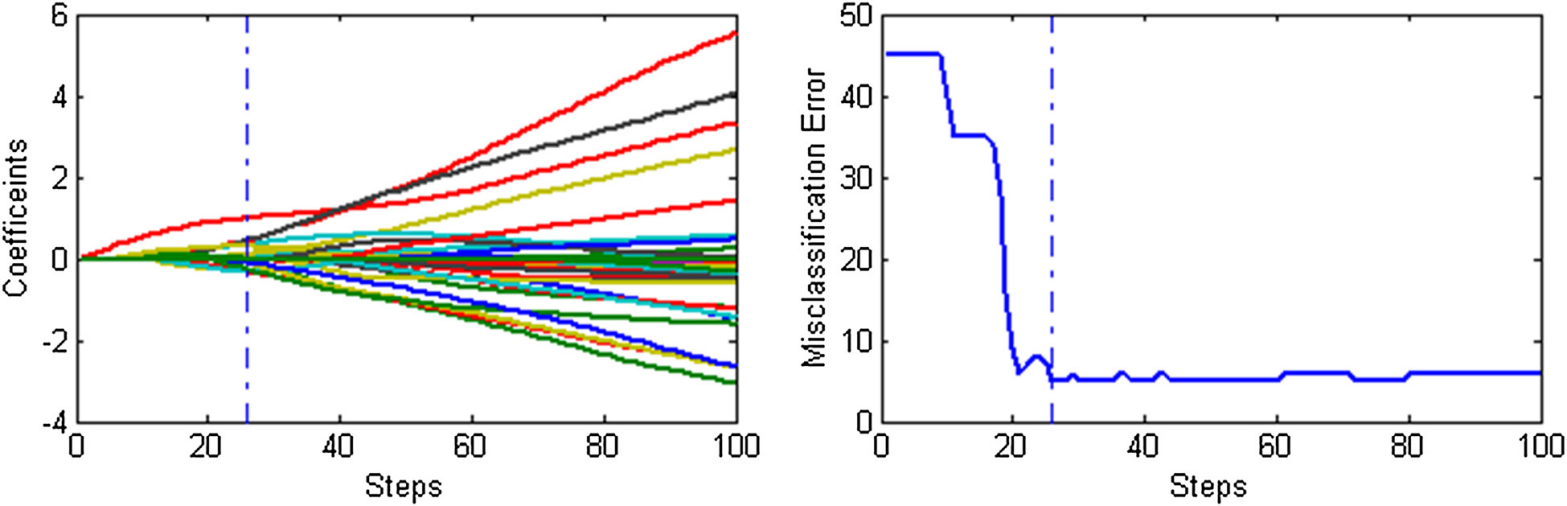
**The results of the sparse logistic regression with the L**_**EN **_**penalty on Prostate dataset.** The solution paths and the gene selection results of the sparse logistic elastic net penalty methods for the Prostate dataset in one sample run.

**Figure 3:**
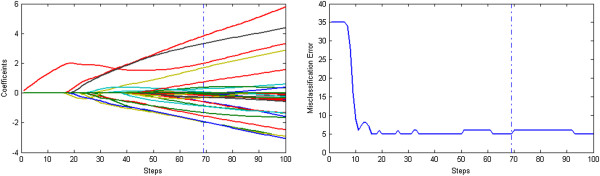
**The results of the sparse logistic regression with the L**_**1 **_**penalty on Prostate dataset.** The solution paths and the gene selection results of the sparse logistic L_1_ penalty methods for the Prostate dataset in one sample run.

Figures 1, 2 and 3 display the solution paths and the gene selection results of the three methods for the Prostate dataset in one sample run. Here the x-axis displays the number of running steps, the y-axis in the left sub-figure is the coefficients measured gene importance and the y-axis in the right sub-figure is the misclassification errors based on the ten-fold cross validation. The optimal results of three methods are shown as vertical dotted lines. Figure 1 indicates that the number of nonzero coefficients (selected genes) of the optimal results obtained by the L_1/2_ method is 5. In contrast, Figures 2 and 3 indicate that the numbers of nonzero coefficients (selected genes) of optimal results obtained by the L_EN_ and L_1_ methods are 37 and 26 respectively. Generally speaking, the penalized logistic regression methods can be successfully applied to the cancer classification problems with high dimensional and low samples microarray data, and our proposed L_1/2_ method achieves better performance especially in gene selection.

### Brief biological analyses of the selected genes

The summaries of the 10 top-ranked informative genes found by the three sparse logistic regression methods for 4 gene expression datasets are shown in Tables 7, 8, 9 and 10 respectively. The genes with star(*) are the most frequently selected genes to construct the classifiers according to the last column of Table 6, and the common genes obtained by each classifier are emphasized with bold. The biologically experimental results proved some genes included in the frequently selected gene sets that produce high classification accuracy rate are mostly and functionally related to carcinogenesis or tumor histogenesis. For example, in Table 7, the most frequently selected gene set of each sparse logistic method for leukemia classification, including cystatin C (CST3) and myeloperoxidase (MPO) genes, that achieve high classification accuracy by the L_1/2_ method, are experimentally proved to be correlated to leukemia of ALL or AML. The cystatin C gene is located at the extracellular region of the cell and has role in invasiveness of human glioblastoma cells. Decrease of cystatin C in the CSF might contribute to the process of metastasis and spread of the cancer cells in the leptomeningeal tissues [23]. The myeloperoxidase gene is taking role in anti-apoptosis process where cancer cells kill themselves [24]. For the colon dataset (Table 9), the most frequently selected gene set of each sparse logistic method includes genes such as guanylate cyclase activator 2B (GUCA2B), myosin, light chain 6, alkali, smooth muscle and non-muscle (MYL6) and Human desmin (DES) genes. These genes are the top 3 significant informative genes ranked by our proposed L_1/2_ method and also selected by Ben-Dor et al. [25], Yang and Song [26] and Li et al. [27]. On the top of these genes lists is guanylate cyclase activator 2B (GUCA2B) gene. Notterman et al. [28] showed that a reduction of uroguanylin might be an indication of colon tumors, and Shailubhai et al. [29] reported that treatment with uroguanylin has a positive therapeutic significance to the reduction in pre-cancerous colon ploys.

**Table 7:** The 10 top-ranked informative genes found by the three sparse logistic regression methods from the Leukaemia dataset

**Rank**	**Gene description**		
	**L**_**1/2**_	**L**_**EN**_	**L**_**1**_
1	**CST3 cystatin C** *	CFD complement factor D (adipsin) *	**CST3 cystatin C** *
2	**MPO myeloperoxidase** *	**CST3 cystatin C** *	CFD complement factor D (adipsin) *
3	**IL8 interleukin 8**	**MPO myeloperoxidase** *	**MPO myeloperoxidase** *
4	GYPB glycophorin B (MNS blood group)	**DNTT deoxynucleotidyltransferase, terminal** *	**IL8 interleukin 8** *
5	**IGL immunoglobulin lambda locus**	TCL1A T-cell leukemia/lymphoma 1A *	**DNTT deoxynucleotidyltransferase, terminal** *
6	**DNTT deoxynucleotidyltransferase, terminal**	**IGL immunoglobulin lambda locus** *	TCL1A T-cell leukemia/lymphoma 1A *
7	LOC100437488 interleukin-8-like	**IL8 interleukin 8** *	**IGL immunoglobulin lambda locus**
8	**LTB lymphotoxin beta (TNF superfamily, member 3)**	ZYX zyxin *	**LTB lymphotoxin beta (TNF superfamily, member 3)**
9	TCRB T cell receptor beta cluster	**LTB lymphotoxin beta (TNF superfamily, member 3)** *	CD79A CD79a molecule, immunoglobulin-associated alpha
10	S100A9 S100 calcium binding protein A9	CD79A CD79a molecule, immunoglobulin-associated alpha	HBB hemoglobin, beta

The genes with star(*) are the most frequently selected genes to construct the classifiers according to the last column of Table 6, and the common genes obtained by L_1/2_ , L_EN_ , L_1_ classifiers are emphasized with bold.

**Table 8:** The 10 top-ranked informative genes found by the three sparse logistic regression methods from the Prostate dataset

**Rank**	**Gene description**		
	**L**_**1/2**_	**L**_**EN**_	**L**_**1**_
1	**SLC43A3 solute carrier family 43, member 3** *	**AMOTL2 angiomotin like 2** *	**USP4 ubiquitin specific peptidase 4 (proto-oncogene)** *
2	**CD22 CD22 molecule** *	**USP4 ubiquitin specific peptidase 4 (proto-oncogene)** *	**CD22 CD22 molecule** *
3	KHDRBS1 KH domain containing, RNA binding, signal transduction associated 1 *	**EIF4EBP2 eukaryotic translation initiation factor 4E binding protein 2** *	**EIF4EBP2 eukaryotic translation initiation factor 4E binding protein 2** *
4	ZNF787 zinc finger protein 787 *	PRAF2 PRA1 domain family, member 2 *	Gene symbol:AA683055, probe set: 34711_at *
5	GMPR guanosine monophosphate reductase *	CACYBP calcyclin binding protein *	**AMOTL2 angiomotin like 2** *
6	**AMOTL2 angiomotin like 2**	Gene symbol:AA683055, probe set: 34711_at *	VSNL1 visinin-like 1 *
7	**EIF4EBP2 eukaryotic translation initiation factor 4E binding protein 2**	VSNL1 visinin-like 1 *	FLNC filamin C, gamma *
8	USP2 ubiquitin specific peptidase 2	**SLC43A3 solute carrier family 43, member 3** *	PRAF2 PRA1 domain family, member 2 *
9	**USP4 ubiquitin specific peptidase 4 (proto-oncogene)**	**CD22 CD22 molecule** *	CACYBP calcyclin binding protein *
10	ACTN4 actinin, alpha 4	TMCO1 transmembrane and coiled-coil domains 1 *	**SLC43A3 solute carrier family 43, member 3** *

The genes with star(*) are the most frequently selected genes to construct the classifiers according to the last column of Table 6, and the common genes obtained by L_1/2_ , L_EN_ , L_1_ classifiers are emphasized with bold.

**Table 9:** The 10 top-ranked informative genes found by the three sparse logistic regression methods from the colon dataset

**Rank**	**Gene description**		
	**L**_**1/2**_	**L**_**EN**_	**L**_**1**_
1	**GUCA2B guanylate cyclase activator 2B (uroguanylin)** *	**GUCA2B guanylate cyclase activator 2B (uroguanylin)** *	**GUCA2B guanylate cyclase activator 2B (uroguanylin)** *
2	**MYL6 myosin, light chain 6, alkali, smooth muscle and non-muscle** *	**MYH9 myosin, heavy chain 9, non-muscle** *	**ATPsyn-Cf6 ATP synthase-coupling factor 6, mitochondrial** *
3	**DES desmin** *	**DES desmin** *	**MYH9 myosin, heavy chain 9, non-muscle** *
4	CHRND cholinergic receptor, nicotinic, delta polypeptide *	**MYL6 myosin, light chain 6, alkali, smooth muscle and non-muscle** *	GSN gelsolin *
5	PECAM1 platelet/endothelial cell adhesion molecule-1 *	GSN gelsolin *	**MYL6 myosin, light chain 6, alkali, smooth muscle and non-muscle** *
6	**ATPsyn-Cf6 ATP synthase-coupling factor 6, mitochondrial**	COL11A2 collagen, type XI, alpha 2 *	COL11A2 collagen, type XI, alpha 2 *
7	ATF7 activating transcription factor 7	**ATPsyn-Cf6 ATP synthase-coupling factor 6, mitochondrial** *	MXI1 MAX interactor 1, dimerization protein *
8	PROBABLE NUCLEAR ANTIGEN (Pseudorabies virus)[accession number:T86444]	ssb single-strand binding protein *	UQCRC1 ubiquinol-cytochrome c reductase core protein I *
9	**MYH9 myosin, heavy chain 9, non-muscle**	Sept2 septin 2 *	**DES desmin** *
10	MYH10 myosin, heavy chain 10, non-muscle	MXI1 MAX interactor 1, dimerization protein *	ZEB1 zinc finger E-box binding homeobox 1*

The genes with star(*) are the most frequently selected genes to construct the classifiers according to the last column of Table 6, and the common genes obtained by L_1/2_ , L_EN_ , L_1_ classifiers are emphasized with bold.

**Table 10:** The 10 top-ranked informative genes found by the three sparse logistic regression methods from the DLBCL dataset

**Rank**	**Gene description**		
	**L**_**1/2**_	**L**_**EN**_	**L**_**1**_
1	**CCL21 chemokine (C-C motif) ligand 21** *	MTH1 metallothionein 1H *	MTH1 metallothionein 1H *
2	HLA-DQB1 major histocompatibility complex, class II, DQ beta 1 *	**MT2A metallothionein 2A** *	**MT2A metallothionein 2A** *
3	**MT2A metallothionein 2A** *	**SFTPA1 surfacant protein A1** *	**CCL21 chemokine (C-C motif) ligand 2** *
4	THRSP thyroid hormone responsive *	TCL1A T-cell leukemia/lymphoma 1A *	**SFTPA1 surfacant protein A1** *
5	**lgj immunoglobulin joining chain** *	ZFP36L2 ZFP36 ring finger protein-like 2 *	POLD2 polymerase (DNA directed), delta 2, accessory subunit *
6	TCL1A T-cell leukemia/lymphoma 1A *	FCGR1A Fc fragment of IgG, high affinity Ia, receptor (CD64) *	**lgj immunoglobulin joining chain** *
7	GOT2 glutamic-oxaloacetic transaminase 2, mitochondrial (aspartate aminotransferase 2) *	**lgj immunoglobulin joining chain** *	MELK maternal embryonic leucine zipper kinase *
8	Plod procollagen lysyl hydroxylase *	TRB2 Homeodomain-like/winged-helix DNA-binding family protein *	CKS2 CDC28 protein kinase regulatory subunit 2 *
9	STXBP2 syntaxin binding protein 2 *	MELK maternal embryonic leucine zipper kinase *	EIF2A eukaryotic translation initiation factor 2A, 65kDa *
10	**SFTPA1 surfacant protein A1** *	**CCL21 chemokine (C-C motif) ligand 2** *	AQP4 aquaporin 4 *

The genes with star(*) are the most frequently selected genes to construct the classifiers according to the last column of Table 6, and the common genes obtained by L_1/2_ , L_EN_ , L_1_ classifiers are emphasized with bold.

In Tables 7, 8, 9 and 10, some genes are only frequently selected by the L1/2 method, but not discovered by the L_EN_ and L_1_ methods. The evidence from the literatures showed that they are cancer related genes. For example, for the colon dataset, the genes cholinergic receptor, nicotinic, delta polypeptide (CHRND) and platelet/endothelial cell adhesion molecule-1 (PECAM1) were also selected by Maglietta R. et al. [30], Wiese A.H. et al. [31], Wang S. L. et al. [32], and Dai J. H. and Xu Q. [33]. These genes can significantly discriminate between non-dissected tumors and micro dissected invasive tumor cells. It is remarkable that apparently (to our knowledge) some discovered genes that have not been seen in any past studies.

On the other hand, from Tables 7, 8, 9 and 10, we found that the most frequently selected genes and their ranking orders by the LEN and L1 methods are much similar compared with those of the L1/2 method. The main reasons are that the classification hypothesis needs not be unique as the samples in gene expression data lie in a high-dimensional space, and both of the LEN and L1 methods are based on the L1 type penalty.

### Construct KNN classifier with the most frequently selected relevant genes

In this section, to further evaluate the performance and prediction generality of the sparse logistic regression with L_1/2_ penalty, we constructed KNN (*k* =3, 5) classifiers using the relevant genes which were most frequently selected by the L_1/2_ penalized logistic regression method. In this experiment, we use the random leave-one-out cross validation (LOOCV) to evaluate the predictive ability and repeat 50 runs.

Table 11 summarizes classification accuracies of four datasets with KNN classifiers with selected genes by our proposed methods. From Table 11, we can see that all the classification accuracies are high than 90%, especially the classification accuracy on the Leukaemia dataset is 98.3%. The KNN classifiers with relevant genes which were selected by the sparse logistic regression with the L_1/2_ penalty can achieve high classification accuracy. The results indicate that the sparse logistic regression with the L_1/2_ penalty can select power discrimination genes.

**Table 11:** **Summary of the results of KNN classifiers using the most frequently selected genes by our proposed L**_**1/2 **_**penalized logistic regression method**

**Methods**	**K-NN(k=3)**	**K-NN(k=5)**
Leukaemia	98.3%	94.4%
Prostate	95.1%	94.2%
Colon	95.1%	90.6%
DLBCL	94.8%	91.2%

## Conclusions

In cancer classification application based on microarray data, only a small subset of genes is strongly indicative of a targeted disease. Thus, feature selection methods play an important role in cancer classification. In this paper, we propose and model sparse logistic regression with the L_1/2_ penalty, and develop the corresponding coordinate descent algorithm as a novel gene selection approach. The proposed method utilizes a novel univariate half thresholding to update the estimated coefficients.

Both simulation and microarray data studies show that the sparse logistic regression with the L_1/2_ penalty achieve higher classification accuracy than those of ordinary L_1_ and elastic net regularization approaches, while fewer but informative genes are selected. Therefore, the sparse logistic regression with the L_1/2_ penalty is the effective technique for gene selection in real classification problem.

In this paper, we use the proposed method to solve binary cancer classification problem. However, many cancer classification problems involve multi-category microarray data. We plan to extend our proposed method to solve multinomial penalized logistic regression for multiclass cancer classification in our future work.

## Competing interests

All authors declare they have no competing interests.

## Authors’ contributions

YL, CL and XZL developed the gene selection methodology, designed and carried out the comparative study, wrote the code, and drafted the manuscript. KSL, TMC, ZBX and HZ brought up the biological problem that prompted the methodological development and verified and provided discussion on the methodology, and co-authored the manuscript. The authors read and approved the manuscript.
